# Cytomegalovirus viral load within blood increases markedly in healthy people over the age of 70 years

**DOI:** 10.1186/s12979-015-0056-6

**Published:** 2016-01-05

**Authors:** Helen M. Parry, Jianmin Zuo, Guido Frumento, Nikhil Mirajkar, Charlotte Inman, Emma Edwards, Mike Griffiths, Guy Pratt, Paul Moss

**Affiliations:** Institute of Immunology and Immunotherapy, University of Birmingham, Vincent Drive, Birmingham, B152TT UK; University of Birmingham Medical and Dental School, Vincent Drive, Birmingham, B15 2TT UK; West Midlands Regional Genetics Laboratories, Birmingham Women’s NHS Foundation Trust, Mindelsohn Way, Edgbaston, Birmingham, B15 2TG UK; University Hospitals NHS Foundation Trust, Birmingham, UK; Charles Darwin Building, Henwick Grove, University of Worcester, Worcester, WR2 6AJ UK

**Keywords:** Cytomegalovirus, Ageing, Monocyte, ddPCR, Lifespan

## Abstract

**Background:**

Cytomegalovirus (CMV) is a highly prevalent herpesvirus, which maintains lifelong latency and places a significant burden on host immunity. Infection is associated with increased rates of vascular disease and overall mortality in the elderly and there is an urgent need for improved understanding of the viral-host balance during ageing.

CMV is extremely difficult to detect in healthy donors, however, using droplet digital PCR of DNA from peripheral blood monocytes, we obtained an absolute quantification of viral load in 44 healthy donors across a range of ages.

**Results:**

Viral DNA was detected in 24 % (9/37) of donors below the age of 70 but was found in all individuals above this age. Furthermore, the mean CMV load was only 8.6 copies per 10,000 monocytes until approximately 70 years of age when it increased by almost 30 fold to 249 copies in older individuals (*p* < 0.0001). CMV was found within classical CD14+ monocytes and was not detectable within the CD14-CD16+ subset. The titre of CMV-specific IgG increased inexorably with age indicating that loss of humoral immunity is not a determinant of the increased viral load. In contrast, although cellular immunity to the structural late protein pp65 increased with age, the T cell response to the immediate early protein IE1 decreased in older donors.

**Conclusion:**

These data reveal that effective control of CMV is impaired during healthy ageing, most probably due to loss of cellular control of early viral reactivation. This information will be of value in guiding efforts to reduce CMV-associated health complications in the elderly.

**Electronic supplementary material:**

The online version of this article (doi:10.1186/s12979-015-0056-6) contains supplementary material, which is available to authorized users.

## Background

Cytomegalovirus (CMV) is one of eight human herpesviruses and maintains a state of lifelong latency within the host following primary infection. CMV is highly prevalent in all parts of the world and infection rates increase with age, with seropositivity estimated between 50 and 95 % in those aged over 5 years [1, 2]. Viral replication is controlled by the development of a strong cellular and humoral CMV-specific immune response and this must be maintained throughout life in order to prevent episodes of clinically significant viral reactivation [3]. The magnitude of the CMV-specific immune response within the blood is very large and higher than has been recorded against other pathogens [4, 5]. Moreover this immune response increases further with age in a phenomenon that has been termed ‘memory inflation’ [6]. This is associated with reduction in the CD4:8 ratio and accumulation of large numbers of late-differentiated memory cells [7]. However, there is now concern that CMV infection can serve to accelerate the development of immune senescence and several studies have shown that CMV seropositivity is associated with a range of clinical problems and increased risk of mortality in older people [8–12].

Although CMV rarely leads to overt clinical problems after primary infection, it is believed that subclinical episodes of CMV reactivation occur frequently during a lifetime but are rapidly controlled by the host immune response [13]. In order to understand more about the mechanisms by which CMV infection may impact on the health of elderly donors it is important to improve understanding of the level of CMV load within the blood and how this is related to specific features of the CMV-specific immune response. This information might then potentially be used to determine the optimal ‘set point’ of viral-host balance and as such serve as an aspiration to achieve within future interventional therapy, for instance with anti-viral or immune modulatory treatment.

The sites of CMV latency include haemopoietic stem cells, monocytes and epithelial cells. However the level of CMV within the blood is very low and conventional PCR assays are almost invariably negative in healthy donors [14–17]. Indeed this is a useful clinical finding, as a positive CMV PCR is generally interpreted as evidence of clinically significant reactivation in immune suppressed donors and can be used to guide anti-viral therapy. However, purification of discrete cell subsets that harbor viral infection, followed by PCR amplification, is one approach that can be taken to increase the sensitivity of viral detection [18]. Self-renewing CD34+ haemopoietic stem cells represent a reservoir for maintaining viral infection and it has been estimated that latent virus is present in 0.01–0.001 % of myeloid progenitor cells within bone marrow [15, 19, 20]. Monocytes are the major mature haemopoietic cellular host for CMV carriage and the frequency of infected cells is thought to be approximately 10 fold lower [21, 22]. Viral latency is maintained during monocytic carriage, whereas lytic viral replication can only arise following differentiation of monocytes into macrophages, due to changes in the pattern of chromatin binding to the immediate early promoter [23–25].

The use of highly sensitive PCR assays increases the frequency of CMV detection and nested PCR offers the advantage of substantial sensitivity but is poorly quantitative. Droplet digital PCR (ddPCR) is a new approach that provides a highly sensitive and direct method for detection of target DNA without the need for developing a ‘standard curve’. ddPCR emulsifies an oil-based PCR reaction into thousands of droplets, each of which then acts as a PCR micro-reaction and increases the chances of a rare event being detected. Using Poisson’s distribution, a direct measurement of the target DNA can then be determined. ddPCR does not therefore rely on any interpretation of rate-based data, as is the case with Q-PCR. The sensitivity and versatility of ddPCR for detection of low copy number events has been shown in several settings and is increasingly part of clinical practice for monitoring mutations levels in malignant disease [26, 27].

We have utilized purification of monocytes combined with droplet digital PCR to permit accurate quantification of the level of CMV within the blood of healthy donors. Our data showed that this provides a highly informative technique to quantify CMV viral load. Moreover they revealed that virus is found infrequently within the blood until the age of around 70 years when it becomes detectable in every donor. We showed that virus is present exclusively within the ‘classical’ CD14+ monocyte fraction and that the increase in viral load correlated with a decrease in the cellular immune response to immediate early protein. These findings have important implications for understanding the biology and clinical complications of CMV infection.

## Results

### Droplet digital PCR can be used for detection of CMV within the monocytes of healthy donors

**Fig. 1:**
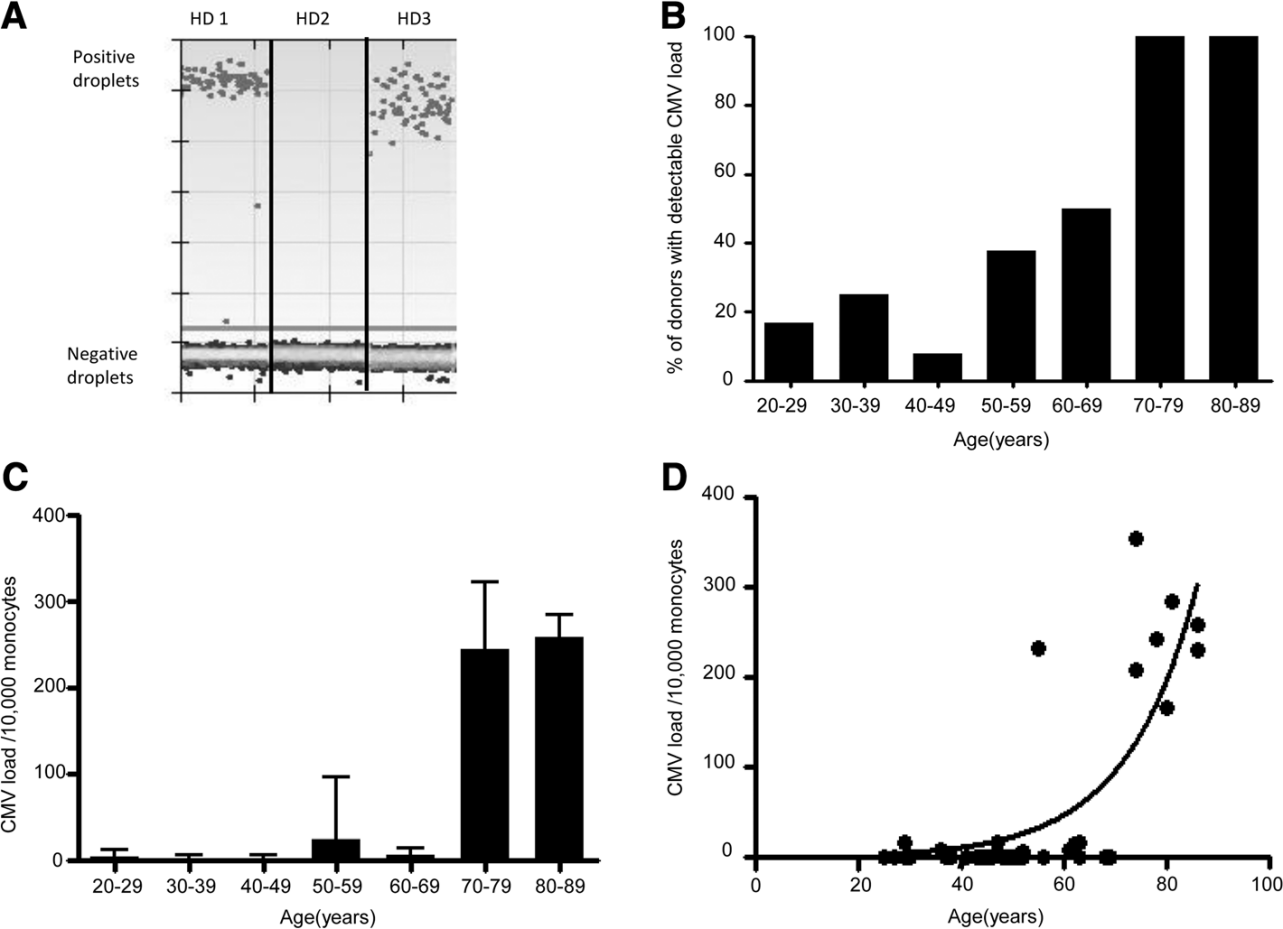
CMV viral load in monocytes increases with ageing. CMV viral load within monocytes increases markedly above the age of 70 years. The DNA of purified CD14+ monocytes was extracted and CMV virus load was detected using droplet digital PCR analysis (ddPCR). **a** Positive droplets were defined as those detectable above the set threshold as shown in healthy donor (HD) 1 and 3. HD 2 had no detectable CMV viral load. **b** The CMV virus load was checked in 44 donors, aged between 20 and 90 years of age. The frequency of donors with detectable latent CMV viral load increases with age. **c** The absolute viral load per 10,000 monocytes was determined by QuantaSoft® software using RRP30 as an internal control. The CMV viral load increases dramatically over the age of 70. **d** The CMV virus load data was modelled with the exponential growth curve to show the CMV viral load doubling time was 9.6 years

Blood samples were obtained from 44 healthy donors between 25 and 86 years of age. CD14+ monocytes were purified by positive selection using magnetic beads and then DNA was extracted using miniprep kits. CMV-specific PCR primers and droplet digital PCR analysis (ddPCR) was then used to determine the number and proportion of CMV-positive droplets within each sample. Positive droplets were defined as those detectable above the set threshold (Fig. 1a) and Poisson’s distribution was used to determine the absolute copy number of the reaction. Using this approach, CMV was detected in 16 of the 44 donors (36 %) and was confirmed in each case by 3 independent runs.

### CMV viral load within monocytes increases markedly above the age of 70 years

When the results were assessed in relation to donor age it was clear that the proportion of donors in whom CMV was detectable increased markedly with age. Specifically, CMV was detected in 9 of 37 (24 %) of donors aged below 70 years whereas a positive test was seen in each of the 7 donors above this age (Fig. 1b). In those aged 20–30 years, only 1 out of 6 donors (16.7 %) had a detectable load, compared to 1 out of 4 (25 %) in 30–40 year olds, 1 out of 13 (7.7 %) in 40–50 year olds, 3 out of 8 (37.5 %) in 50–60 year olds and 3 out of 6 (50 %) in 60–70 year olds.

The absolute quantification of CMV viral load in relation to monocyte number was then determined by QuantaSoft® software in order to generate a value of ‘viral load per 10,000 monocytes’. In those donors in whom CMV was detectable by ddPCR, the absolute viral load varied markedly from 3 copies per 10,000 monocytes to 353 copies per 10,000 monocytes, a range of 117 fold. This value was also found to increase with age, again with a marked increase observed in donors aged over 70 years (kruskal-wallis *p* = 0.0005). The mean viral load in donors aged below 70 years was 8.6 copies per 10,000 monocytes (SD 38), with a 29-fold increase in those over the age of 70, where the mean viral load was 249 copies per 10,000 monocytes (SD 59) (Fig. 1c). Modelling of the data with an exponential growth curve showed that the CMV viral load doubling time was 9.6 years (R^2^ = 0.64) (Fig. 1d).

### The increase in CMV viral load with age is confirmed through the use of quantitative PCR

We next used a second method in order to confirm the observation of an increase in CMV load within monocytes in relation to age. Quantitative PCR (Q-PCR) was applied to the same sample cohort using an average plasmid series dilution, which produced an R^2^ value of 0.983 (*p* < 0.0001) (Additional file 1: Figure S1A). Using triplicate runs, Q-PCR was sensitive down to a single copy of plasmid CMV per reaction. This same series of diluted plasmids was then verified using ddPCR and the absolute copy number of CMV was calculated through QuantaSoft® software. ddPCR was again capable of detecting 1 copy of CMV per reaction indicating that results generated from ddPCR and Q-PCR correlated very strongly (R^2^ = 0.995; *p* = <0.0001) (Additional file 1: Figure S1B).

We then used Q-PCR to assess CMV load within the 44 samples of monocyte DNA from healthy donors. Thirteen of the 16 samples that were found to be positive by digital PCR were also detected as positive by Q-PCR (81 %). Twenty-seven out of the 28 samples that were negative by ddPCR were also negative by Q-PCR and only one was reported positive by Q-PCR. The correlation between the two techniques using 13 results that were positive by both methods had a co-efficient of determination of R^2^ = 0.626 (*p* = 0.0013). The Q-PCR technique also revealed a pronounced increase in viral load in association with age (Additional file 1: Figure S1C).

### CMV viral load is detectable within CD34+ haemopoietic cells and is focused within ‘classical’ CD14+ monocytes

**Fig. 2:**
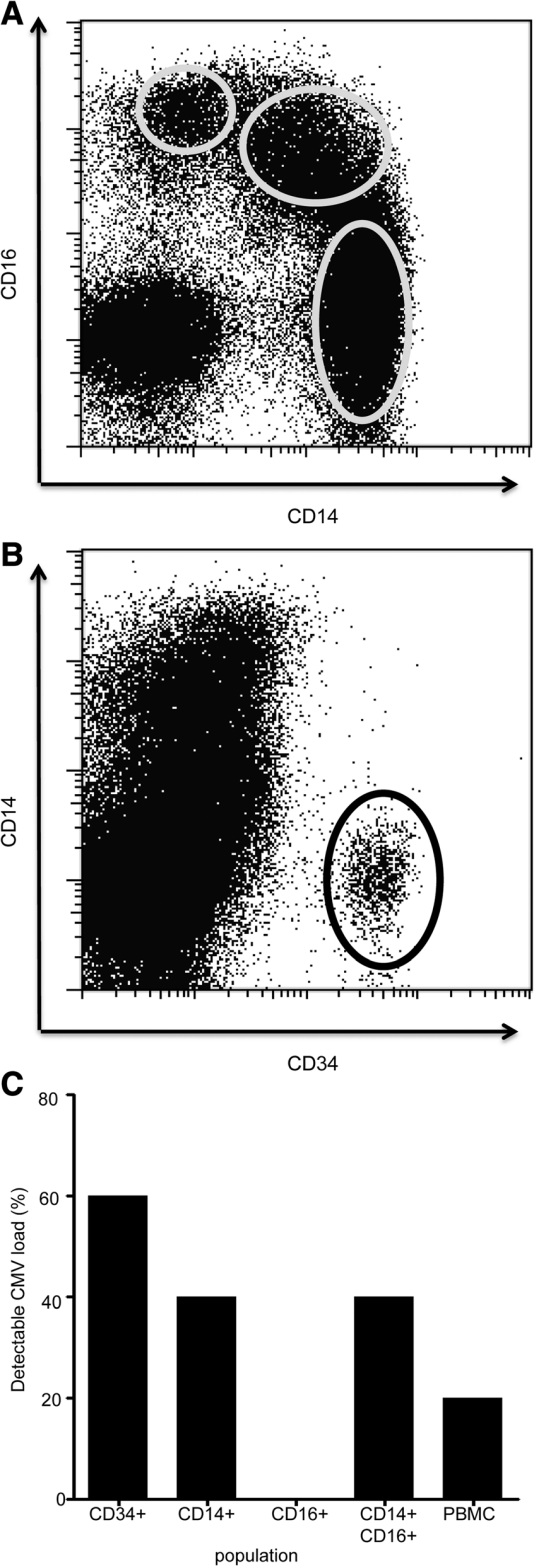
CMV viral load is detectable in CD14 positive monocytes. CMV viral load is detectable within CD34+ haemopoietic cells and is focused within ‘classical’ CD14+ monocytes. DNA from CD34+, CD14 + CD16-, CD14 + CD16+ and CD14-CD16+ populations were used to detect CMV viral load using ddPCR. **a** Representation of flow plots used for selecting monocyte populations based on CD14+ and CD16+ antibody staining. **b** Representation of flow plots used for selecting CD34+ cells. **c** The frequency of detection of CMV viral load was compared between the different subpopulations

In order to determine the distribution of CMV load within different cells of the myeloid lineage we then went on to perform ddPCR on purified CD34+ haemopoietic stem cells and individual monocyte subsets. CD14 and CD16 expression can be used to subdivide the major subpopulations of monocytes into CD14 + CD16-, CD14 + CD16+ and CD14-CD16+ subsets (Fig. 2a). Fractions of G-CSF-mobilised peripheral blood were obtained from five haemopoietic stem cell donors and cells were sorted into CD34+ stem cells and monocytic subsets (Fig. 2b). CMV was detected in 3 of these 5 samples through the use of both ddPCR and Q-PCR. Interestingly, within the monocyte subsets, CMV was found only within classical monocytes with a CD14+ phenotype whereas the CD14-CD16+ subset was entirely negative by both assays (Fig. 2c).

### The titre of the CMV-specific IgG response increases with age

**Fig. 3:**
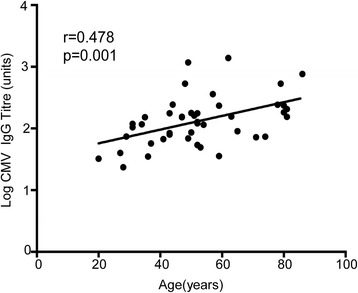
CMV igG titre increases with ageing. CMV-specific IgG antibody titre increased substantially during ageing, with a three-fold increase in titre between the ages of 20 and 80 years

Our previous data showed an increase in CMV load within the peripheral blood during healthy ageing, so we next went on to determine the magnitude of the CMV-specific immune response in association with age. In particular, we assessed if the increase in viral load might result from a reduction in the magnitude of virus-specific immunity or if the immune response might actually increase as a response to the increased level of virus in the blood. We therefore determined the CMV-specific IgG titre using a quantitative ELISA assay against viral lysate. Interestingly, antibody titre increased substantially during ageing with a three fold increase in titre between the ages of 20 and 80 years (mean titre 65.1 in donors aged 20 years compared to 232 in those aged over 70); r = 0.473; *p* = 0.001). This increase in titre developed gradually during ageing and therefore had a different pattern to the quite dramatic elevation in viral load that was seen only after the age of 70 years (Fig. 3).

### The CMV-specific T cell response to pp65 also increases with age whereas recognition of IE-1 is reduced in elderly donors

We next went on to assess the magnitude of the T cell immune response against CMV within the study cohort. Specifically, we focused on the cellular responses to pp65 and immediate early 1 (IE-1), which are two of the most immunodominant components of the CMV proteome. Several previous studies have reported the substantial magnitude of the CMV-specific T cell immune response within peripheral blood and have also identified that this can increase further with age [28, 29].

**Fig. 4:**
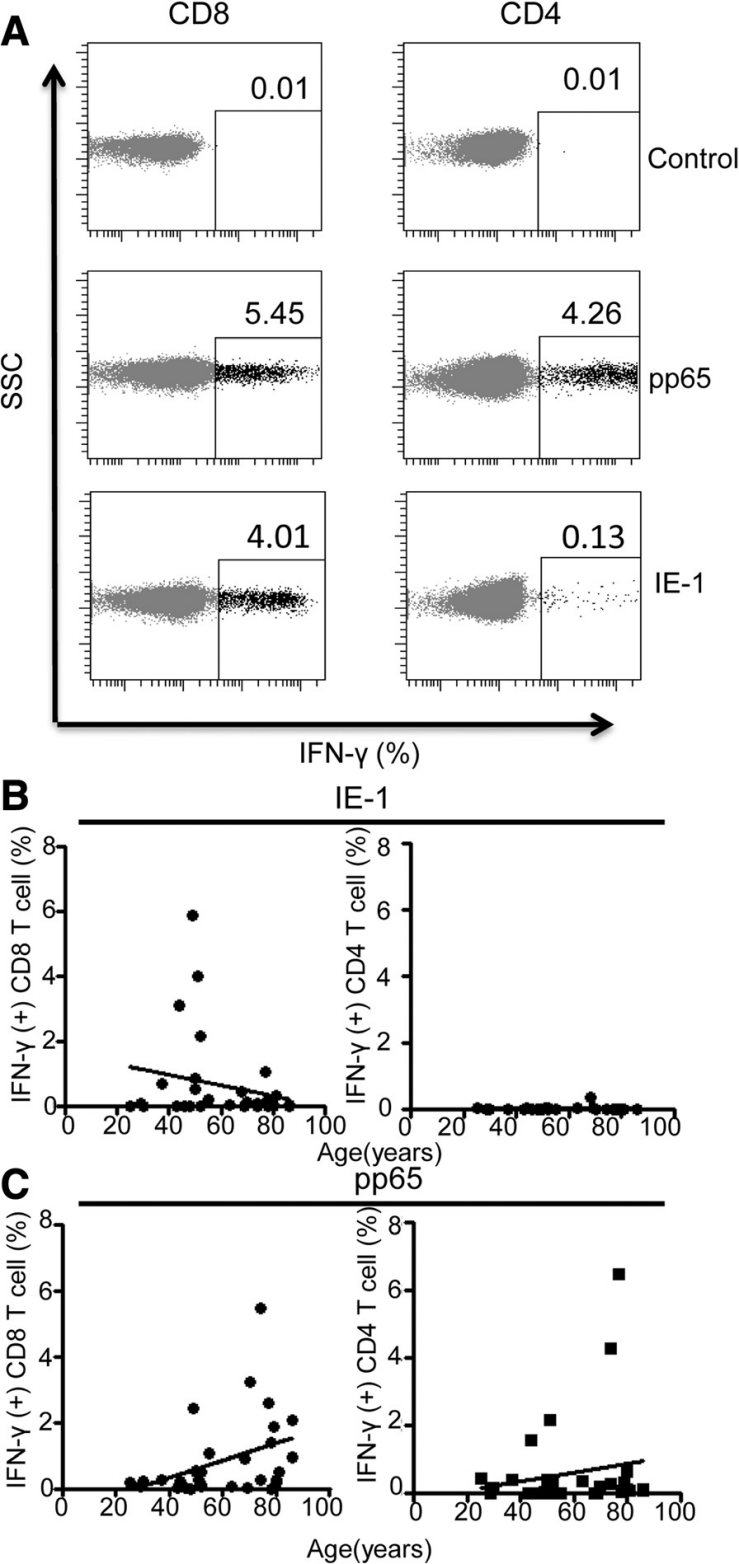
The T cell response to the immunodominant CMV protein pp65 increases with age. T cells were stimulated with peptide pools containing either pp65 or IE-1 and the IFN-g release by peptide-specific CD4 and CD8 T cells was then determined. **a** Representation of the flow plots for IFN-g response to pp65 and IE-1 peptide stimulation. **b** Correlation of the T cell response to IE-1 peptides against age demonstrated a peak in people aged 50–60 years followed by a decreased response in older donors. The CD4+ T cell response to IE-1 peptide was small but remained relatively constant across all ages. **c** Both CD4 and CD8 T cell response to pp65 increase with ageing

T cells were stimulated with peptide pools containing immunodominant epitopes from either pp65 or IE-1 and the IFN-γ release by peptide-specific CD4 and CD8 T cells was then determined by flow cytometry (Fig. 4a). The magnitude of the pp65-specific CD8+ T cell immune response ranged from 0 to 5.5 % of the CD8+ T cell pool, with a median value of 0.28 %. This value was over two-fold higher in those donors with detectable CMV viral load within monocytes compared to donors in whom the ddPCR was negative, but this did not reach statistical significance (0.5 vs 0.23 % respectively; *p* = 0.087). The pp65-specific T cell response also increased markedly with age, from a median value of 0.17 % in 20–30 year olds to 1.17 % in those aged greater than 70 years (r^2^ = 0.146, *p* = 0.03). A positive correlation with age was also noted following pp65 stimulation of CD4+ T cells, although this did not reach statistical significance.

In contrast to pp65, the frequency of CD8+ T cells recognizing IE-1 initially increased with age, but then peaked in people aged 50–60 years and actually decreased in older donors (median 0.01 % <50 years; 0.7 % 50–60 years and 0.1 % >70 years old; R^2^ = 0.05; *p* = 0.244). The CD4+ T cell response to IE-1 peptide stimulation was of small magnitude but remained relatively constant across each age group, with a median frequency of 0.01 % (Fig. 4b).

In summary, the magnitude of the cellular immune response to the structural late protein pp65 increased with age, whilst the CD8+ T cell response to IE-1 peaked at the age of 50–60 years and reduced thereafter.

## Discussion

Cytomegalovirus infection has been associated with a variety of health problems in elderly people and there is increasing interest in the mechanisms that underlie this association. A key determinant in this regard will be greater understanding of the balance of the viral load and the host immune response during healthy ageing. In this study we report, for the first time, that the level of cytomegalovirus viral load within the blood increased markedly in elderly people.

A novel feature of our work was the use of digital droplet PCR to provide an accurate quantitative measure of latent viral DNA. Previous methods for detection of CMV from monocytic DNA generally relied on nested PCR techniques, which made quantification challenging and also raised substantial problems with reproducibility [30]. Quantitative PCR is far more accurate but relies on interpretation of the cycle threshold of a sample against a known calibration standard. This is restricted by the lower limit of detection of the standards and the rate of amplification, which can vary between different PCR runs. In contrast, digital PCR provides an absolute quantification and avoids these limitations. Our analysis included a direct comparison of ddPCR and Q-PCR and, as expected, we observed an extremely high concordance between the two technologies. However ddPCR was found to offer superior sensitivity and reliability of detection. Seventeen samples were found to be positive by either ddPCR or Q-PCR, 16 of these by ddPCR and 14 by Q-PCR.

Our work was performed using DNA isolated from monocytes, which are established as the most important haemopoietic site of viral latency [31, 32], and which in murine infection also serve to disseminate viral infection to distal sites such as salivary gland [33]. The first interesting finding was the observation that CMV was detectable in only a minority of donors, as 64 % of people remained negative by ddPCR despite the presence of chronic infection as confirmed by CMV-specific IgG positivity. Indeed, in younger people below the age of 50 years, the detection of CMV load in the blood was uncommon, being observed in only 13 % of donors tested. The lower limit of detection provided by ddPCR in our assay was for a single copy of virus within the total reaction volume (20 μl) and as such a negative result indicated absent or extremely low levels of virus. This low level carriage may reflect a lower intrinsic probability of viral reactivation in younger donors but is perhaps more likely to reflect the consequence of effective immune surveillance of viral replication in younger individuals.

The frequency of viral detection increased markedly with each decade above the age of 50 years to 37.5 and 50 % and finally became positive in every donor who was older than 70. Interestingly the amount of viral DNA detected within the blood also increased substantially with age with a 29 fold increase observed between donors aged less than 70 and those over this age. The use of nested PCR also detected viral DNA within the majority of healthy elderly donors [18]. These data indicate that a gradual impairment in the ability to control CMV load within blood starts around the age of 50 years and then deteriorates markedly beyond the age of 70. Importantly, our work did not address the number of CMV copies within individual monocytes, which has previously been shown to vary between 2 and 13 copies per cell [15]. Thus, it remains uncertain if ageing is associated with an increase in the number of viral copies within each infected monocyte or if there is an increase in the proportion of infected cells.

We were also interested to use the sensitivity of ddPCR to examine the presence of CMV within specific subsets of the myelo-monocytic lineage. CD14 and CD16 can be used to delineate three major subclasses of monocyte [34], classical monocytes (CD14 + CD16-) which account for >70 % of peripheral monocytes and are important for innate immunity. These, together with intermediate monocytes (CD14 + CD16+) have more phagocytic properties than their non-classical CD14-CD16++ counterparts [35]. Our data suggested that CMV was not detectable within CD14-CD16+ monocyte cells, a finding which is in contrast to murine CMV, where CD16+ monocytes have been shown to exhibit higher levels of CMV latency than CD14+ cells [33].

We were also interested to compare levels of CMV load within haemopoietic precursors of the terminally differentiated monocytic lineage. As such we isolated CD34+ haemopoietic stem cells from G-CSF mobilized blood donations. CMV was detected in 3 of these 5 samples, which is comparable to a previous report of 8 positive samples out of 12 using an alternative PCR methodology [15]. This suggests that viral DNA may either pass selectively into cells that differentiate into the monocytic lineage or that some degree of viral replication occurs during myelopoiesis in order to sustain viral loads during the periods of cellular proliferation prior to monocyte formation.

Importantly, the ddPCR assay detects the level of viral DNA but does not assess the level of infectious virion. CMV was not detected by ddPCR within plasma samples taken from donors identified to have a positive monocyte viral load (n = 10). This indicated that viral DNA was retained within cells, with no evidence of production of extracellular virus (data not shown).

The direct measurement of viral DNA is a critical component in efforts to understand the balance of viral load and host immune response during natural CMV infection, an ambition that has been achieved so effectively for the study of HIV using RNA load. However, it is also important to correlate these values with assessment of the immune response to the virus.

**Fig. 5:**
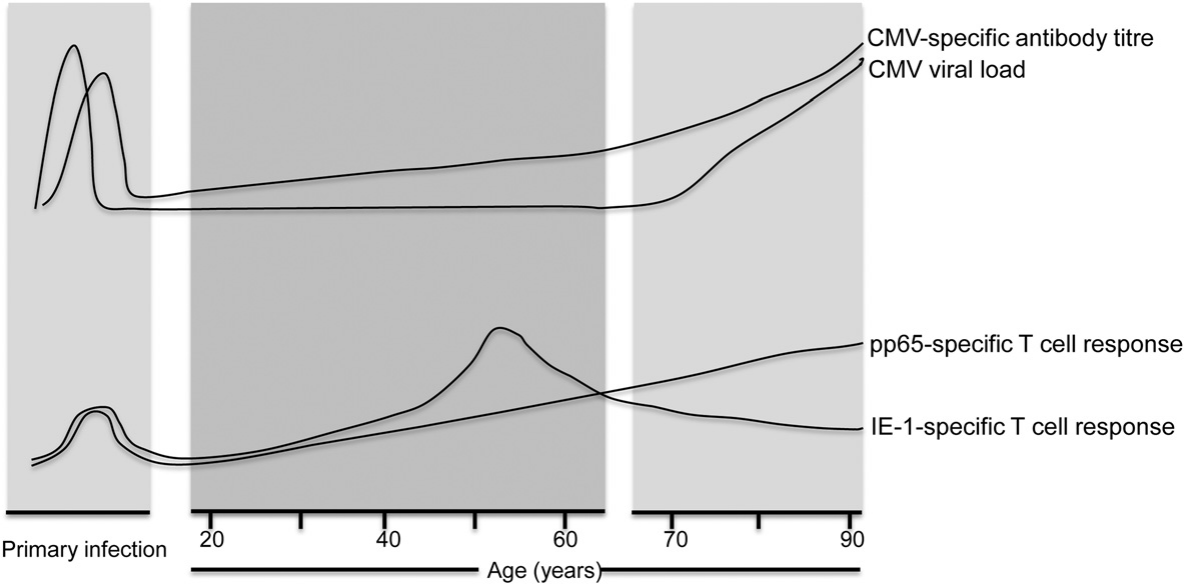
An overview of the parallel changes in viral load and immunity during the lifetime of an individual infected with Cytomegalovirus. Schematic representation of the relative changes in the peripheral blood viral load as well as humoral and T cell immune response to CMV over the human life-course. Primary infection is shown as occurring during childhood, the most common age of infection. CMV viral load and CMV-specific IgG antibody titre increases during ageing. CD8 T cell responses to IE-1 peptides peaked in people aged 50–60 years and then decreased in older donors, while T cell response to pp65 peptides increase with ageing

Figure 5 gives a schematic overview of the parallel changes in immunity and viral load that arise following CMV infection over a lifetime. We observed that the humoral immune response to CMV increased steadily and markedly during ageing. Total immunoglobulin levels are known to fall with ageing and so our increase in CMV-specific antibody might appear somewhat surprising, but has in fact been reported previously [36, 37]. It is likely that episodes of subclinical viral reactivation serve to boost CMV-specific immunity and indeed this accumulation of CMV-specific immune memory may suppress the development of heterologous immune responses, which is a likely contributing factor towards immune senescence. Nevertheless these observations suggest that impairment in humoral immunity is not a major contributory factor towards the increase in viral load with ageing.

Studies have shown that the T cell immune response to CMV increases markedly with age such that the virus-specific CD8+ T cell response can come to dominate the CD8+ T cell repertoire in some donors [3, 38]. This profile of ‘memory inflation’ is also seen in murine CMV infection and is believed to be driven by recognition of viral peptides on non-haemopoietic cells. Relatively little is known about the specific profile of CMV proteins that drive CD8+ T cell expansion during ageing, although the importance of structural proteins such as pp65 are well documented [3, 29]. Indeed, the presence of CMV DNA within monocytic cells has been correlated with increased pp65-specific T cell immunity in elderly donors [18].

Our work also observed an expansion of pp65-specific T cells in relation to ageing, with an 8.4 fold increase between the youngest and most elderly donors and may reflect an incremental accumulation to recurrent subclinical episodes of viral reactivation. However, assessment of the T cell recognition of peptides derived from the immediate early protein IE-1 revealed an increase in middle aged individuals, with reduced recognition in those of an advanced age. This observation supports previous work, which has reported no significant increase in the IE-1 specific immune response with ageing [39].

It is currently unclear why IE-1 responses appear to wane with age. An important factor may relate to the frequency of T cell exposure to IE-derived epitopes, as these are presented to T cells repeatedly during the early period of viral replication and serve to elicit a cellular immune response that can prevent the late stages of viral infection before late viral proteins such as pp65 are produced. As such IE-1-specific T cells are exposed to very high levels of antigen stimulation and may be more susceptible to functional exhaustion than those directed against late-stage viral proteins such as pp65 [40]. However the explanation for the differing T cell responses to IE-1 and pp65 with ageing remains unclear and will require larger cross sectional and longitudinal study.

The concept that ageing leads to an alteration in the balance between viral load and the host immune response during chronic viral infection is supported by a variety of clinical and immunological observations. Herpes zoster, which represents reactivation of varicella zoster virus (VZV), is more common in the elderly and VZV titres increase with age, a pattern also reported for Epstein Barr Virus [41, 42]. Finally, levels of the persistent and highly prevalent Torque tenovirus are also known to increase markedly with ageing [43].

## Conclusion

In conclusion, these data reveal the delicate balance that has evolved between chronic CMV infection and the host immune response and indicate that this symbiosis can break down during ageing, where an increase in CMV viral load occurs as the attritional effects of chronic surveillance and the impact of immune senescence become more apparent. It is likely that increased understanding of the clinical importance of chronic viral infection on human health will become an important health consideration in future years.

## Methods

### Healthy donors

Forty-four CMV positive healthy donors (confirmed by CMV ELISA) were recruited for study between the ages of 25 and 86 (mean 50.5). Healthy individuals below the age of 65 were recruited from The University of Birmingham, whilst those over 65 were recruited as part of the ongoing Birmingham 1000 elder’s cohort which recruits elderly healthy donors from the local community. Following a 50 ml blood donation, plasma and PBMCs were extracted over a ficoll density gradient and stored at −160 °C. PBMCs were used for functional T cell studies and monocyte extraction whilst plasma was used for CMV ELISA testing.

Five peripheral blood stem cell donors who had received G-CSF mobilization were also recruited and aliquots of PBMC stored at −160 °C prior to defrosting and extraction of myeloid cell subsets.

### Extraction of cell subsets: CD14, CD34 and CD16

Enrichment of CD14, CD16, CD34 and dual positive CD14/16 cells from stem cell donations were sorted by flow cytomety (MoFLow sorter, BDBiosciences, Oxford, UK). Following defrosting, cells were washed in PBS and labeled for 15 min at 4° with LIVE/DEAD Fixable red dead cell stain kit (Life technologies, UK), PE anti-CD34 (BDBiosciences), anti-CD56 FITC (BDBiosciences), anti-CD14 FITC (BDBiosciences) and anti-CD16 Pe-Cy7 (Biolegend, San Diego, USA), prior to a further wash and sorting. For extraction of CD14 positive cells from healthy donor PBMC, positive selection using CD14 magnetically labeled beads was used and an average enrichment found to be 98.73 % (SD 0.39) by flow cytometry (Miltenyi Biotec, Surrey, UK). DNA extraction was then performed on the enriched cell populations according to the protocol for GenElute Mammalian Genomic DNA miniprep kit (Sigma-Aldrich, St. Louis, MO USA) and DNA concentration and purity checked using the Nanodrop 2000 (Thermo Scientific, Waltham, MA, USA).

### CMV plasmid controls for standard curve generation

Human CMV HHV5 kit for Q-PCR amplification of glycoprotein B was purchased and used for all CMV PCR reactions within this work (PrimerDesign, Southampton, UK). Using the provided plasmid control, reconstituted aliquots were stored at −20 °C. Plasmid dilutions were then prepared fresh for Q-PCR to validate detection of the ddPCR assay and were diluted to produce the following copies per reaction: 50000, 10000, 2500, 500, 250, 100, 50, 10, 5 and 1.

### Droplet digital PCR

Using the QX100 droplet digital PCR system (Bio-rad, Pleasanton, CA), a reaction mixture consisting of 5ul of either CD14 positive DNA (10 ng/ul) or plasmid standard made up to a volume of 8ul with PCR grade water, 10ul of 2 × ddPCR supermix for probes (Bio-rad), 1ul of reconstituted FAM labeled CMV primer and probe (Primer Design) and 1ul of HEX labeled RPP30 copy number assay for ddPCR (Bio-Rad) were loaded into a disposable plastic cartridge for droplet generation (Bio-Rad). Seventy microlitre of droplet generation oil (Bio-Rad) was also added before loading the cartridge into the droplet generator (Bio-Rad). After droplet generation, the sample was loaded into a 96 well PCR plate (Eppendorf, Hamburg, Germany) and PCR amplification carried out using the T100 thermocycler (Bio-Rad). PCR conditions consisted of 10 min at 95 °C, prior to 40 cycles at 94 °C for 30 s and 60 s at 60 °C and a finally 1 cycle at 10 min at 98 °C, ending at 12 °C. After amplification the plate was loaded onto the droplet reader (Bio-rad) and results analysed by QuantaSoft® software (Bio-Rad) to give the number of virus copies per ul of PCR reaction. A positive and negative control was used in each experiment, which also verified the consistency of droplet amplitude, and a well consisting solely of water was also included. Results were obtained in triplicate. As each mammalian cell contains 2 copies of RPP30, the absolute quantification of RPP30 was divided by two in order to determine the actual cell number. The CMV viral load was then divided by this figure to obtain the CMV load per cell.

### Q-PCR

Using the 7500 Real Time PCR system (Applied Biosystems, California, USA), a PCR reaction mixture consisting of 5ul of standard plasmid or 5ul of CD14 positive DNA (10 ng/ul) made up to a volume of 9ul with PCR grade water, together with 10ul of 2 × Taqman Universal mastermix II with no UNG (Applied Biosystems) and 1ul of FAM labeled CMV primer and probe were loaded into a 96 well PCR plate (Eppendorf, Hamburg, Germany). PCR amplification consisted of 2 min at 95 °C, prior to 50 cycles at 95 °C for 10 s and 60 s at 60 °C. A positive and negative control plus water well were included in each experiment and the standard curve repeated in triplicate and averaged. Samples were only considered positive if present in triplicate.

### Enzyme-linked immunosorbent assay (ELISA) testing for CMV IgG titre

As described by Kilgour et al, CMV ELISA testing (University of Birmingham, Birmingham UK) was used to ascertain participant’s CMV status. Briefly, mock and viral lysate were used to coat 95 well plate overnight at 4 °C**.** Using plasma from 3 CMV positive donors, a standard was prepared and added to the plate in a 1 in 4 serial dilution, alongside 1ul of healthy donor samples. After 1 h incubation at room temperature, the plate was washed and anti-IgG horseradish peroxidase conjugated secondary antibody (Southern Biotech, Alabama, USA) added to each well and incubated for a further 1 h in the dark, RT. After repeat wash steps, 100 μL of TMB ELISA peroxidase substrate (Rockland Immunochemicals, Pennsylvania, USA) was added and incubated for 10 min in the dark, RT. To stop the reaction, 100 μL of 1 mM HCl was added, prior to reading on an ELISA plate reader at 450 nm [44].

### CMV functionality testing

10^6^ PBMC were resuspended in 500 ul RPMI plus 10 % FCS and incubated with either 10 ul Peptivator EI-1, 10 ul Peptivator pp65 (Miltenyi Biotech, Bergisch Gladbach, Germany), 10 ul SEB (Sigma-Aldrich), or 10 ul RPMI. 0.5 ul of Brefeldin A was then added to all samples (Bio Legend) and incubated over night at 37 °C in 5 % CO_2_. Afterwards, cells were washed and stained with viability dye-APC (Thermo Fisher Scientific) following manufacturer’s instructions. After two washes, surface staining was performed, using anti-CD8 FITC, anti-CD4 PE-Cy7, anti-CD3 APC-Cy7 (BDBiosciences). Cells were then washed, fixed in 4 % paraformaldehyde (Sigma-Aldrich), washed again, and permeabilized with 0.5 % saponin (Sigma-Aldrich). After washing, cells were stained with anti-IFNγ PE (Miltenyi Biotech), washed and analysed using a FacsCanto II flow cytometer (BDBiosciences).

### Statistical analysis

One way ANOVA (Kruskal-Wallis test) and post hoc Dunn’s testing was used to compare CMV viral load with age. A growth exponential curve was used to assess the CMV load doubling time and linear regression used to assess the relationship between CMV copies (log_10_) and the age of participants. For the standard dilution series ran in triplicate, the results were log transformed log_10_ and Pearson’s correlation co-efficient was used to compare the quantitative agreement between ct value and plasmid copy number. Linear regression was used to examine the relationship between ddPCR copy number reading and that of the plasmid dilution and also to compare Q-PCR copy number with that of ddPCR in healthy donor samples. Pearson’s correlation co-efficient was used to examine the relationship between CMV IgG titre and age. For comparison of age against functional T cell responses against Spearman’s correlation co-efficient was used. Mann-Whitney testing was used to compare IFN-y responses in T cells following peptide stimulation in those with detectable viral load compared to those without. All analysis was performed using Prism version 6.0, Graphpad software, San Diego, USA.

## Additional file

Additional file 1: Figure S1. A comparison of droplet digital PCR and QPCR for CMV viral load. (A): Classic quantitative PCR (Q-PCR) was calibrated using a series dilution of plasmid standards for CMV. The Ct value was correlated with copy number in each dilution. (B): The serially diluted standard plasmids were then assessed by ddPCR and the absolute copy number obtained was correlated with the expected CMV copy number. (C): Q-PCR was used to assess CMV load within the 44 samples of monocyte DNA from healthy donors. The CMV copy number of the 13 donors that were positive by both methods (Q-PCR and ddPCR) were then correlated. (PDF 381 kb)
