# Supplementary figures and images for: Cytomegalovirus viral load within blood increases markedly in healthy people over the age of 70 years
Source: Immun Ageing. 2016 Jan 5;13:1. doi: 10.1186/s12979-015-0056-6 (PMC4700608; doi:10.1186/s12979-015-0056-6)

A

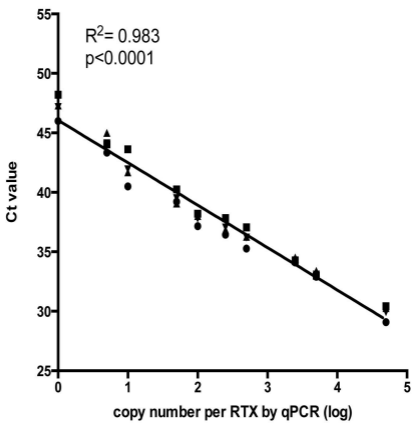

B

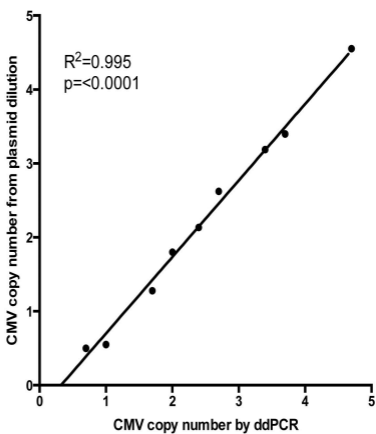

C

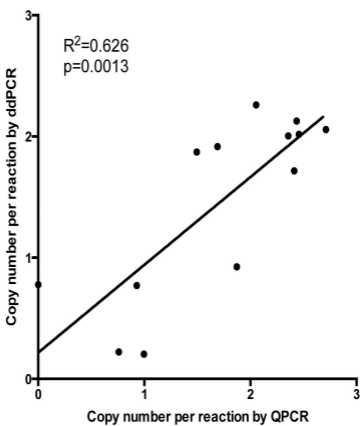

Supplement: Additional file 1: Figure S1. — A comparison of droplet digital PCR and QPCR for CMV viral load. (A): Classic quantitative PCR (Q-PCR) was calibrated using a series dilution of plasmid standards for CMV. The Ct value was correlated with copy number in each dilution. (B): The serially diluted standard plasmids were then assessed by ddPCR and the absolute copy number obtained was correlated with the expected CMV copy number. (C): Q-PCR was used to assess CMV load within the 44 samples of monocyte DNA from healthy donors. The CMV copy number of the 13 donors that were positive by both methods (Q-PCR and ddPCR) were then correlated. (PDF 381 kb) [file 12979_2015_56_MOESM1_ESM.pdf]
